# Atomically Resolved Defect-Engineering Scattering Potential in 2D Semiconductors

**DOI:** 10.1021/acsnano.4c02066

**Published:** 2024-06-26

**Authors:** Hao-Yu Chen, Hung-Chang Hsu, Jhih-Yuan Liang, Bo-Hong Wu, Yi-Feng Chen, Chuan-Chun Huang, Ming-Yang Li, Iuliana P. Radu, Ya-Ping Chiu

**Affiliations:** † Graduate School of Advanced Technology, National Taiwan University, Taipei 10617, Taiwan; ‡ Department of Physics, National Taiwan University, Taipei 10617, Taiwan; § Taiwan Semiconductor Manufacturing Company, Hsinchu 30078, Taiwan; ∥ Institute of Physics, Academia Sinica, Taipei 115201, Taiwan; ⊥ Institute of Atomic and Molecular Sciences, Academia Sinica, Taipei 106319, Taiwan

**Keywords:** Transition metal dichalcogenides, Atomic defect engineering, Intervalley quasiparticle interference, Phase shift, Scanning tunneling microscopy

## Abstract

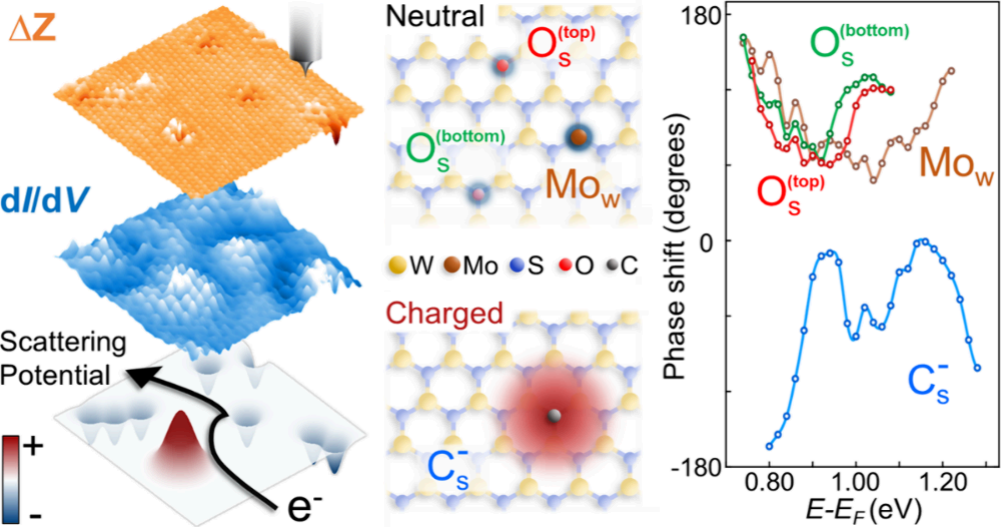

Engineering atomic-scale defects has become an important strategy for the future application of transition metal dichalcogenide (TMD) materials in next-generation electronic technologies. Thus, providing an atomic understanding of the electron–defect interactions and supporting defect engineering development to improve carrier transport is crucial to future TMDs technologies. In this work, we utilize low-temperature scanning tunneling microscopy/spectroscopy (LT-STM/S) to elicit how distinct types of defects bring forth scattering potential engineering based on intervalley quantum quasiparticle interference (QPI) in TMDs. Furthermore, quantifying the energy-dependent phase variation of the QPI standing wave reveals the detailed electron–defect interaction between the substitution-induced scattering potential and the carrier transport mechanism. By exploring the intrinsic electronic behavior of atomic-level defects to further understand how defects affect carrier transport in low-dimensional semiconductors, we offer potential technological applications that may contribute to the future expansion of TMDs.

## Introduction

Defects in semiconductors are essential for transport and optoelectronic device technology. In particular, for the atomically thin two-dimensional (2D) transition metal dichalcogenides (TMDs), the low-dimensional quantum confinement makes them even more susceptible to electronic defect structure.^1^ In view of the effectively targeted control and utilization of electronic defect characteristics, there are growing efforts to extend the functional progress of defect engineering in TMDs to alter their physicochemical applications.^2−4^

Most of the intrinsic vacancies on the transition metal and chalcogen atom sites naturally originate from the synthetic deposition or exfoliation process of TMDs.^5^ This leads to unpaired valence electrons or dangling bonds and introduces additional deep in-gap states that could trap the mobile carriers, resulting in unexpected consequences on electronic transport.^6−8^ For the purpose of avoiding the trapping event, the doping strategy in TMDs with substitutional elements has been widely used to improve the electronic performance of TMDs.^9−11^ However, even though the substitutional and the original atoms have similar electron configurations to achieve relatively stable electronic structures, there are still discrepancies between their atomic details that can be revealed in the electron scattering events. In general, the electronic defect scattering potential (*U*_*Scatter*_) has been empirically approximated by the composition of the short-range substitution-induced potential (*U*_*sub*_) and the additional long-range Coulomb potential (*U*_*Coul*_).^12−14^ The former *U*_*sub*_ is the intrinsic potential difference attributed to the atomic discrepancy between the substitutional and the original atom.^15^ The latter *U*_*Coul*_ is based on the dopant type of substitutional atom relative to the original atom.^12,13^ Therefore, without the extra charge injected by the substitutional atom that contributes *U*_*Coul*_ in the defect scattering potential (*U*_*Scatter*_ = *U*_*sub*_ + *U*_*Coul*_), the *U*_*sub*_ term will play an important role in the electronic scattering event and impact the carrier transports. Intriguingly, the recent theoretical works predict that the substitution from specific atoms could also bring the coexisting *U*_*sub*_ and *U*_*Coul*_ to compensate for each other, leading to enhanced mobility under high carrier concentration.^15,16^ Thus, providing a comprehensive understanding of the defect scattering potential is indeed a crucial key point for the future transport performance of TMD applications.

However, the diverse types of defects often coexist and mix in one material, making it challenging in macroscopic transport measurements to clarify their respective impacts and contributions to a specific interest. The direct insight into how the mobile carrier couples with different types of atomic defects remains elusive, which limits our understanding of the electron–defect interactions in the TMD systems. Scanning tunneling microscopy and spectroscopy (STM/S) are atomically resolved tools for sensitively probing the defect structure and its electronic properties in real space. To further investigate electron–defect scattering, quasiparticle interference (QPI) manifesting the standing wave near the atomic defect is a suitable approach that originates from elastic scattering of the electronic quantum state. The QPI standing waves can be obtained by the spatially resolved STS mapping technique, which has presented the energy-momentum landscape in TMD systems in previous work.^17−19^ Moreover, observing the spatial QPI standing waves can also reveal the phase shift (ϕ_*shift*_) between the incident and scattered electronic waves, which depends on the different energies of the electronic states and the characteristics of the defect *U*_*Scatter*_ values.^20−24^ Thus, observing the ϕ_*shift*_ variation of spatial QPI standing waves with different types of atomic defects can directly provide insights into how different defects’ *U*_*Scatter*_ values influence the details of electron–defect interactions.

In this work, we present the combination of low-temperature STM/S (LT-STM/S) and the QPI technique to offer a direct approach to the scattering event between the electronic states and atomic defects in TMDs. There are distinct types of substitutional atomic defects observed on the tungsten disulfide (WS_2_) surface, offering the opportunity to investigate the different defect *U*_*Scatter*_ values interacting with electronic states. In addition, the energy-dependent ϕ_*shift*_ variations of QPI standing waves are observed and quantified in this work, leading to direct comparisons between types of defect *U*_*Scatter*_ and their respective impacts on carrier transport.

## Result

### Atomic Defects and QPI Observation on the Tungsten Disulfide Surface

**Figure 1:**
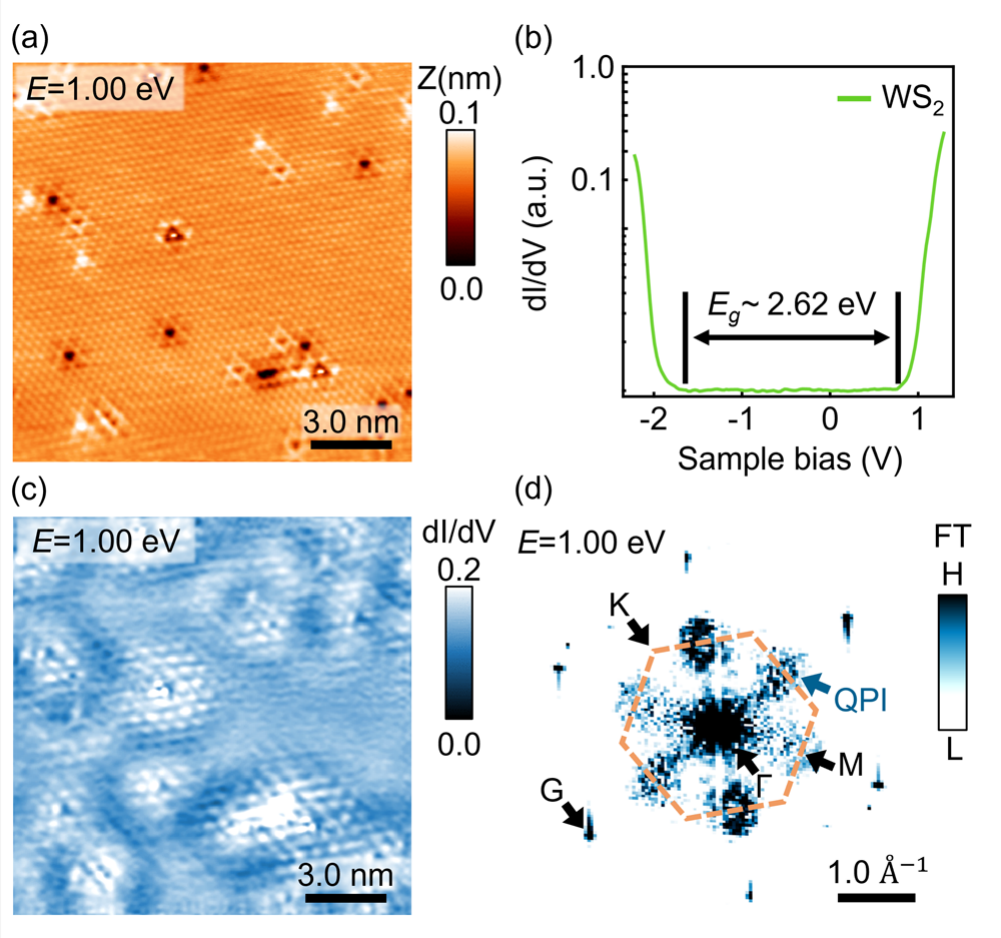
STM topographic image and the spectroscopy of WS_2_/HOPG. (a) The 15 × 15 nm^2^ atomically resolved STM images of WS_2_ at an energy level of 1.00 eV. (b) STS spectrum on the intrinsic defect-free WS_2_ surface, revealing an energy gap of 2.62 ± 0.02 eV. (c) The d*I*/d*V* image corresponds to the scanning region on the topographic image in (a) and reveals the QPI patterns in real space. (The 1 × 1 atomic arrangement is removed by an FT filter.) (d) The 1.00 eV FT-STS map from (c) shows the reciprocal lattice **G**-point and the orange-dashed hexagon is the 1st Brillouin zone of WS_2_. Obvious spots from the defect-induced QPI pattern are observed near the **M**-point.

The growth of WS_2_ synthesized by the chemical vapor deposition (CVD) method on a highly ordered pyrolytic graphite (HOPG) substrate was performed for the STM/S measurements in this work. In Figure 1a, the atomically resolved STM image recorded at +1.00 V sample bias shows the defective surface of WS_2_, revealing the distinct types of atomic defects. To further probe the conduction electronic states representing the mobile carriers, Figure 1b shows the differential conductance STS spectrum measured on the defect-free region of the WS_2_ sample surface at 77 K in UHV. The conduction band minimum (CBM) is located at 0.73 ± 0.02 eV, and an electronic energy gap (*E*_g_) is 2.62 ± 0.02 eV for the WS_2_/HOPG system, consistent with the previous work.^19^ The information on the substrate-induced moiré patterns and whether moiré is related to the formation of QPI is discussed in Supporting Information 1. Above the CBM energy level, these atomic defects induce the local conduction electronic state scattering, leading to the spatially distributed wave patterns in the d*I*/d*V* image, shown in Figure 1c. (The 1 × 1 atomic arrangement is removed by filter.) The spatially distributed wave patterns observed near each atomic defect presenting the (2 × 2)-like period can be analyzed from the 2D Fourier transform (2D-FT) of the d*I*/d*V* image in Figure 1d. In addition, the orange-dashed hexagon-like shape in Figure 1d indicates the first Brillouin zone (BZ) of WS_2_, and the apparent spots are located on the **M**-point due to the (2 × 2)-like period around each defect in real space. The origin of this (2 × 2)-like wave distribution can be attributed to the electronic states scattering between Q valleys at the conduction band of ML-WS_2_ due to the well-separated valley properties closer to the CBM in 2D-TMD systems,^25,26^ which also has been confirmed previously.^17,19^ The quantitative band structure analysis of the energy-dependent QPI wavevector and the large spin-splitting characteristic at the Q valley in ML-WS_2_ are discussed in Supporting Information 2.

### Distinct Types of Defects and Their QPI Standing Wave Variations

**Figure 2:**
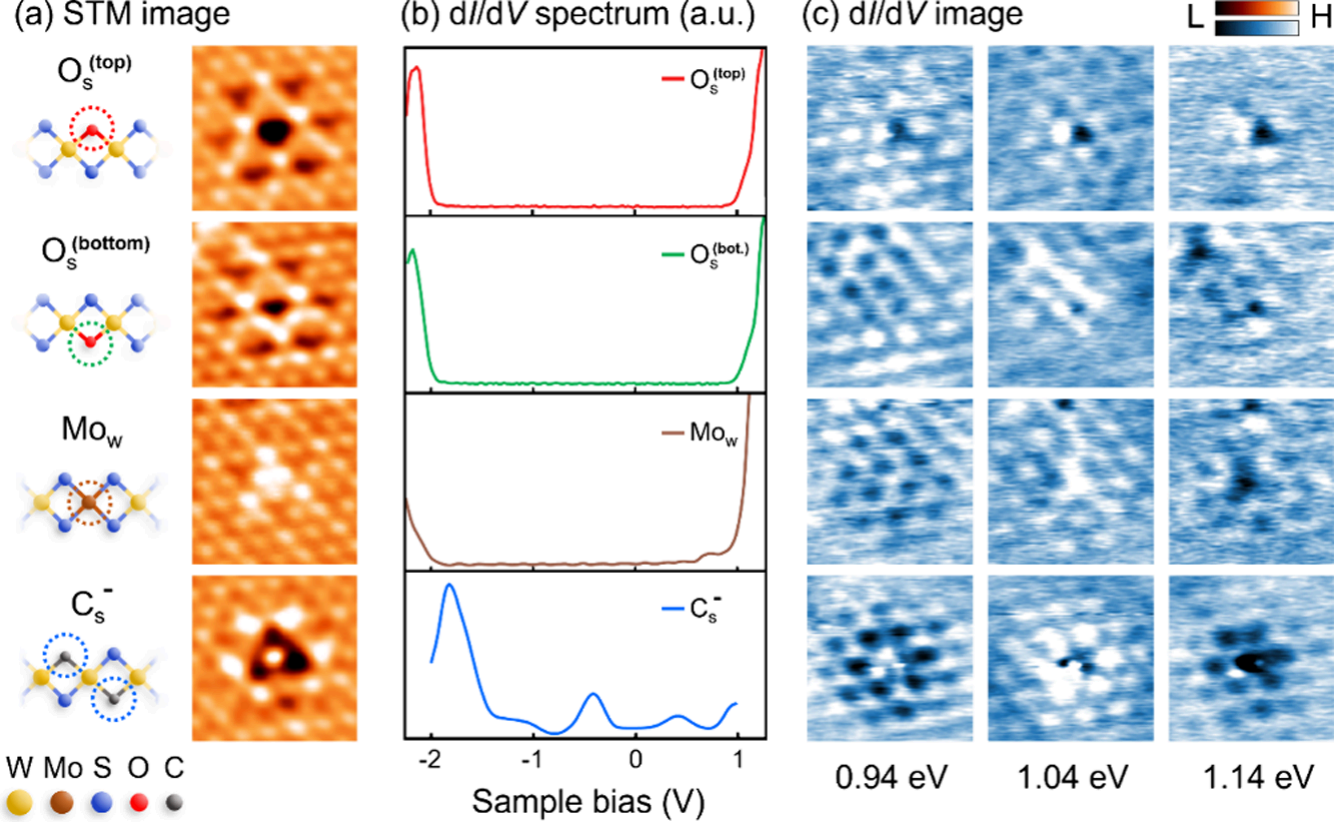
STM image, d*I*/d*V* image, and spectroscopy of different types of atomic defects. (a) The local 2 × 2 nm^2^ STM image of defects at an energy level of 1.00 eV, including O_s_^(top)^, O_s_^(bottom)^, Mo_w_, and C_s_^–^. (b) The d*I*/d*V* spectra of O_s_^(top)^, O_s_^(bottom)^, Mo_w_, and C_s_^–^. Only C_s_^–^ presents the obvious in-gap states near the Fermi level (at sample bias V = 0). (c) The local 3 × 3 nm^2^ STS maps at energy levels of 0.94, 1.04, and 1.14 eV, which correspond to STM images in (a) and directly reveal the energy-dependent QPI pattern variations in real space.

Figure 2 presents the local STM images, d*I*/d*V* images, and STS spectra in terms of four substitutional types of defects observed on the WS_2_ surface in this work. The substitutional defects on the tungsten and sulfur atom sites naturally originated from the synthetic deposition process, and other elements from the precursor impurity or exposure under the atmosphere could also participate in the substitution events. These substitutional defects can be qualitatively identified from previous STM/S work,^6,7,27−29^ including the oxygen substituting on the top and bottom layers of the sulfur sites (O_s_^(top)^ marked in red; O_s_^(bottom)^ marked in green), the molybdenum substituting on the tungsten site (Mo_w_ marked in brown), and the negatively charged carbon substituting on sulfur sites (C_s_^–^ marked in blue). The corresponding defect number densities in Supporting Information 3 provide quantitative information to characterize the presence of these common defect types. However, with reference to previous research work,^7^ other substitution defects such as vacancies on sulfur sites (S_vac_) and chromium substitution on tungsten sites (Cr_w_) have also been found in CVD-grown WS_2_. In order to preserve the original characteristics of the samples and to practically explore the feasibility of future applications, no other sample handling procedures, such as annealing, were carried out when the samples were loaded into the UHV chamber.^30^ Due to this situation, S_vac_ and Cr_w_ were hardly found in our WS_2_ samples with the estimated defect number density < 10^10^ cm^–2^ with a total statistical area of about 200 × 200 nm^2^. Figure 2a,b presents their corresponding atomic structures and electronic characteristics, respectively. However, it should be noted that only C_s_^–^ presents the obvious in-gap states near the Fermi level (at sample bias V = 0), while the others show the absence of in-gap states, consistent with their corresponding STS properties in previous work.^6,7,28^ In Figure 2c, the local d*I/*d*V* images from 0.94 to 1.14 eV show the highly resolved QPI standing wave patterns near these defects, leading to the direct comparison of the QPI patterns’ appearance between these distinct types of defects. The QPI patterns of these defects can be regarded as the composition of the QPI wavefront along the direction corresponding to the QPI spots located on the **M**-point in Figure 1d, showing the distribution discrepancy of the standing wave crest and trough relative to their defect center. Significantly, combining the defect center in Figure 2a and QPI standing wave patterns in Figure 2c, only Mo_w_ presents the 3-fold symmetry distribution of the electronic topography and the QPI wavefront, while the other defect types show the 6-fold symmetry behavior. It could be attributed to the different defect electronic characteristics or the discrepancy of the geometrical positions between the tungsten and sulfur atom sites, leading to the additional symmetry-dependent scattering rules.^31^

In general, the spatial distribution of a standing wave can be mathematically described by its wavevector and phase. Due to the domination of the intervalley interference between the two electronic states, the wavevector of QPI periods in this work is nearly invariant and only varied between the spin-up and spin-down Q valleys, as demonstrated in previous work.^17,19^ Thus, the variations of the QPI standing waves in Figure 2c can be directly attributed to the phase-dependent behaviors. Therefore, by comparing the d*I*/d*V* image from 0.94 to 1.14 eV, the QPI standing wave patterns of Mo_w_ and C_s_^–^ obviously reveal their phase variation. For the O_s_^(top)^ and O_s_^(bottom)^, the behavior of their QPI variations are quite similar, but the intensity of the QPI standing wave near O_s_^(bottom)^ is weaker due to the scattering event occurring at the bottom sulfur layer of WS_2_.^32^ Both QPI standing wave intensities of O_s_^(top)^ and O_s_^(bottom)^ are almost degraded at energy level 1.14 eV in Figure 2c, compared to the QPI wave patterns of C_s_^–^ and Mo_w_ still apparent at this high energy level. However, the phase-dependent variations cannot be revealed from reciprocal space analysis and can be observed only through real-space measurements.

### Spatial Analysis of the Energy-Dependent QPI Phase Variations

**Figure 3:**
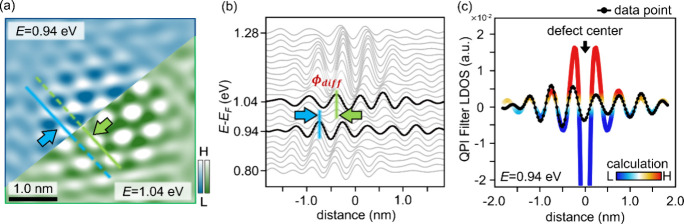
QPI enhanced d*I*/d*V* image, QPI standing wave evolution, and the fitting calculation. (a) The QPI pattern enhanced 4 × 4 nm^2^ d*I*/d*V* image of C_s_^–^ at an energy level of 0.94 and 1.04 eV. The blue and green half-figures on the corner indicate the wavefront of the QPI pattern distribution at 0.94 and 1.04 eV to show the energy-dependent QPI phase difference. (b) The energy-dependent landscape constructed by recording the continuous variation of the QPI standing waves nearby C_s_^–^ in real space with the energy level 0.80 to 1.28 eV (per 0.02 eV interval). The blue and green lines directly indicate the phase difference (ϕ_*diff*_) at 0.94 and 1.04 eV. (c) The fitting results (red-blue gradient color) and the experimental data of C_s_^–^ (black curve), giving the calculated ϕ_*shift*_ = −13° ± 10.2° at energy level 0.94 eV.

Figure 3 presents the spatial analysis of the energy-dependent phase variations, where the QPI patterns near the atomic defects are enhanced by using the 2D-FT filter. Figure 3a directly compares QPI patterns of the C_s_^–^ at energy levels 0.94 and 1.04 eV, indicated by blue and green frames, respectively. The result shows the phase difference (ϕ_*diff*_) between these two energy levels, indicated by the change in the QPI wavefront, as shown by the solid line in each image. In addition, by extracting the line profile of the QPI pattern along its wavefront propagation at different energy levels ranging from 0.80 to 1.28 eV (in 0.02 eV intervals), the energy-dependent landscape is constructed to reveal the continuous variation of the QPI pattern near defect C_s_^–^, as shown in Figure 3b.

**Figure 4:**
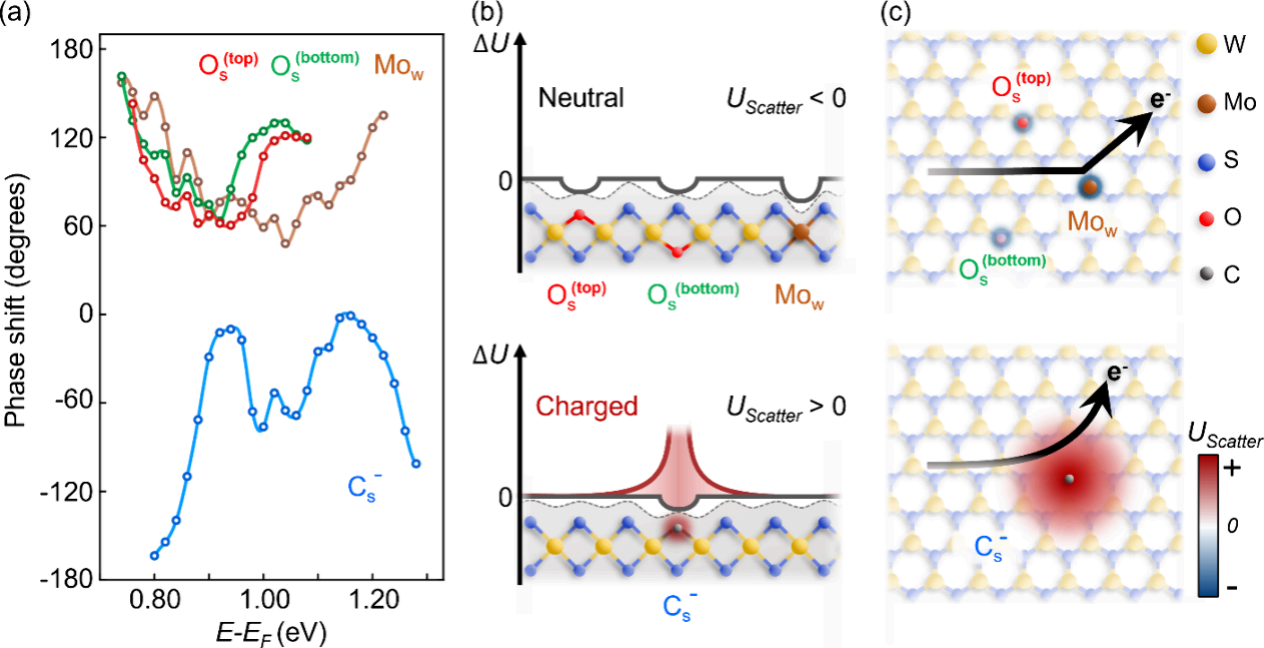
Energy-dependent phase shift variation of the QPI standing wave and the defect scattering potential. (a) The energy-dependent phase shift diagram of O_s_^(top)^, O_s_^(bottom)^, Mo_w_, and C_s_^–^. The C_s_^–^ presents the negative degree tendency of the phase shift; in contrast, the other types of defects present the opposite behavior. (b) The schematic diagram of the defect scattering potential in the side view of WS_2_ classifies O_s_^(top)^, O_s_^(bottom)^, Mo_w_, and C_s_^–^. (c) The schematic diagram of the top view of WS_2_ illustrates the electronic defect scattering event near O_s_^(top)^, O_s_^(bottom)^, Mo_w_, and C_s_^–^.

The theoretical approach to describe the local density of states (LDOS) based on QPI standing waves near a point defect in d*I*/d*V* image can be given by the approximation of the 2D electrons gas system,^20,21^ as the following equation:
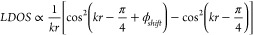
1where *k* is a wavenumber of the standing wave and ϕ_*shift*_ is the phase shift quantified by how the scattered electronic waves differ from the incident waves. Moreover, based on the discussion of the effects of tip potentials in Supporting Information 4, it is suggested that the phase behavior (ϕ_*shift*_) of the QPI standing wave is mainly dominated by the defect scattering potential in the material. Thus, based on eq 1, Figure 3c shows the fitting curve (red-blue gradient color) and the experimental data of C_s_^–^ (black curve), giving the calculated ϕ_*shift*_ = −13° ± 10.2° at the energy level 0.94 eV. The calculation provides an excellent fit for distances greater than 0.3 nm from the defect center. However, the fitting result at the region closer than 0.3 nm to the defect center is relatively challenging due to the complex defect LDOS and the tip-height variation across the defect site.^24^ In addition, the energy-dependent landscape of phase shift variation (Δϕ_*shift*_) can be constructed and quantified by the same analysis process for all defect types at different energy levels in Figure 4a. (See the detailed fitting results in Supporting Information 5.)

## Discussion

### Attractive and Repulsive Defect Scattering Potential

This work presents energy-dependent Δϕ_*shift*_ from different types of substitutional defects in TMDs, which reveals the electronic scattering mechanism involving distinct defect scattering potentials (*U*_*Scatter*_). Comparing the energy-dependent Δϕ_*shift*_ diagram in Figure 4a, only C_s_^–^ presents the variation of ϕ_*shift*_ in the negative degree quadrant, while the other types of defects display the opposite tendency. This discrepancy directly corresponds to the opposite behavior of the QPI wavefront propagation in/outward relative to the defect center with the increasing energy level between the defect C_s_^–^ and other types of defects. In addition, the positive and negative signs of the ϕ_*shift*_ corresponds to the attractive and repulsive defect *U*_*Scatter*_, respectively.^20,21^ To further examine the attractive and repulsive properties of the distinct defect *U*_*Scatter*_, it is necessary to consider both *U*_*sub*_ and *U*_*Coul*_ associated with them. The former refers to the intrinsic atomic differences between the substitutional atom and original atom, while the latter refers to the Coulomb potential resulting from the *n*–*p* type doping of the substitutional atom.^12,13^ As a result, the combination of positive and negative values of *U*_*sub*_ and *U*_*Coul*_ can give rise to four different composition conditions, forming different total values of defect *U*_*Scatter*_ in electronic scattering events.

### Comparisons between the Substitutional and Original Atom

Considering defect types in this work, it is important to note two fundamental comparisons between the substitutional and original atoms: (1) the number of valence electrons and (2) the core electron density. (1) Among these substitutional atoms, only carbon has fewer valence electrons than does the sulfur atom. On the other hand, oxygen and molybdenum have the same number of valence electrons as sulfur and tungsten, respectively. Therefore, as a *p*-type dopant, C_s_^–^ can introduce the additional charge states with its obvious in-gap state properties as the STS results, differing from the neutral properties of O_s_^(top)^, O_s_^(bottom)^, and Mo_w_ without the in-gap states.^6^ As a result, C_s_^–^ acts as an acceptor to accumulate electrons and produce positive *U*_*Coul*_ to repel the electronic states in the scattering process. In contrast, the neutral defect presents no *U*_*Coul*_, and their scattering events are dominated by their *U*_*sub*_. (2) Next, we consider that both the substitutional and original atoms have the same valence electron number to participate in the WS_2_ chemical bonding, leading to the discussion of the core electron discrepancy between them. Thus, for one substitutional atom with more core electrons, it is equivalent to bringing the slightly positive *U*_*sub*_ to the increased core electron density near the defect center. The incident electron penetrating deeper into the defect center could be more challenging, similar to its experiencing the local repulsive potential. In contrast, the lower core electron density will make the incident electron’s scattering closer to the defect center, similar to its experiencing the local attractive potential. Therefore, carbon, oxygen, and molybdenum generally have fewer core electrons than their original atoms (sulfur and tungsten). This means that all types of defects in this work should have the negative value of the attractive *U*_*sub*_, consistent with the smaller atom/ionic radius generating a locally short-range attractive potential in previous theoretical work.^15^ Thus, for O_s_^(top)^, O_s_^(bottom)^, and Mo_w_, they only have a negative *U*_*sub*_ that contributes to their total attractive defect *U*_*Scatter*_ < 0, as their schematic defect *U*_*Scatter*_ diagrams show in Figure 4b. On the other hand, based on the opposite ϕ_*shift*_ behavior in Figure 4a, it is suggested C_s_^–^ should have a larger positive *U*_*Coul*_ that compensates the smaller negative *U*_*sub*_ to form a total repulsive defect *U*_*Scatter*_ > 0, as shown in Figure 4b. Finally, our results verified that the positive/negative sign of the ϕ_*shift*_ corresponds to the attractive/repulsive defect *U*_*Scatter*_, consistent with previous works.^20,21,33,34^ In addition, the electron scattering events from neutral defects, such as O_s_^(top)^, O_s_^(bottom)^, and Mo_w_, are dominated by the short-range attractive *U*_*sub*_, and the electron scattering event from negatively charged C_s_^–^ is dominated by the long-range repulsive *U*_*Coul*_, as seen in the schematic diagram in Figure 4c.

### Impacts on Carrier Mobility in the Electron–Defect Scattering

Next, we compare the maximum magnitude of Δϕ_*shift*_ obtained in Figure 4a. It shows that C_s_^–^ (∼180°) has the highest value, followed by Mo_w_ (∼130°) and O_s_^(top)^ ≈ O_s_^(bottom)^ (∼110°). This comparison is consistent with their cutoff energy levels, which is the highest energy level allowing for detecting the QPI pattern in this work. Above the cutoff energy, the QPI pattern amplitude decays significantly, making it challenging to analyze and extract the ϕ_*shift*_. At such a high energy level, the *U*_*Scatter*_ of the O_s_^(top)^, the O_s_^(bottom)^, and the Mo_w_ defects gradually become a tiny perturbation term and lose their influence on the scattering event with large kinetic energy of the electron. In the case of C_s_^–^, its cutoff energy at 1.28 eV is far from the CBM of WS_2_ and is gradually dominated by the complex electronic states in the band structure,^26^ which causes the loss of Q–Q′ intervalley scattering. Moreover, the maximum magnitude of Δϕ_*shift*_ represents how intensely the defect *U*_*Scatter*_ induces the electronic scattering event.^13,15^ Based on the charge carrier transport mechanics, the electron scattering rate from the impurity is one of the critical factors in determining carrier mobility in the semiconductor. The large magnitude of Δϕ_*shift*_ with both strong repulsive and attractive defect *U*_*Scatter*_ leads to an increased scattering rate and thus reduces carrier mobility.^15^ Therefore, in terms of electronic carrier transport, the extent to which different defects may affect the mobility degradation is C_s_^–^ > Mo_w_ > O_s_^(top)^ ≈ O_s_^(bottom)^.

Inherently, it is reasonable that C_s_^–^ induces the most severe scattering event due to the reduced dielectric screening in 2D-TMDs making the Coulomb interaction more influential.^35^ In addition, the STS result of C_s_^–^ shows that it only presents the in-gap states, which is generally considered to reduce mobility due to electron trapping and losing transport.^36^ However, C_s_^–^ is actually a practical example where its *U*_*Scatter*_ is partially compensated by opposite values of *U*_*sub*_ and *U*_*Coul*_, which directly confirms the possibility for specific doping atoms to reduce its scattering potential.^15^ Thus, one should consider the opportunity that doping engineering integrates the suppressed scattering events from compensated defect potential, ultimately leading to improved mobility. For other neutral defects, substitution on the tungsten site as Mo_w_ results in more degradation of carrier transport compared to substitution on the sulfur site as O_s_. This could be attributed to the *d*-orbitals of tungsten contributing more dominantly to the conduction band, which brings greater differences in additional orbital hybridization than sulfur.^25,37^ However, O_s_^(top)^ and O_s_^(bottom)^ are the most abundant atomic defect, with the orders of numbers greater than Mo_w_ in standard TMD synthetic deposition processes.^38^ Even though they bring the absence of in-gap states, the scattering event still happens with them and impacts the mobile carrier. To minimize additional scattering events, it is suggested that reducing the number of vacancies produced during TMD synthesis or repairing the defect with suitable atoms to reduce *U*_*Scatter*_ is the primary solution to improve carrier transport. This work not only gives insight into the detailed electron–defect interaction with distinct defect *U*_*Scatter*_ at the conduction band but also provides the opportunity to further approach the hole transport by negative STM sample bias in *p*-type semiconductors. Furthermore, our finding also directly supports the targeted defect engineering, such as *n*- to *p*-type doping substitution,^39,40^ impurity repairing,^41,42^ and synthetic deposition improvement,^38^ to achieve the high functional performance of future electronic TMDs applications.

## Conclusion

In conclusion, the achievement of combining LT-STM and the QPI technique to map intervalley quantum interference near the types of atomic defects in TMDs is demonstrated in this work. We observe the distinctive behavior of the QPI standing waves from different types of defects, including O_s_^(top)^, O_s_^(bottom)^, Mo_w_, and C_s_^–^. Furthermore, quantifying the energy-dependent phase variation of the QPI standing wave reveals the detailed electron–defect interaction between the substitution-induced scattering potential and carrier transport mechanism. By exploring the intrinsic electronic behavior of atomic-level defects and then understanding how the defects affect carrier transport in low-dimensional semiconductors, our approach offers potential technological applications and research developments that may contribute to the future development of the TMD.

## Methods

### Sample Preparation

For the experiments, we investigated tungsten disulfide (WS_2_) by low-temperature STM (LT-STM) grown on a highly ordered pyrolytic graphite (HOPG) substrate using a typical chemical vapor deposition process (CVD). Tungsten oxide (WO_3_, Sigma-Aldrich, 99.995%) powders and HOPG substrate are placed at the center and downstream of the furnace, and sulfur (S, Sigma-Aldrich, 99.99%) powders are placed on the upper stream with another temperature control. During the growth, the chamber was kept at 10 Torr and 900 °C (sulfur with 120 °C) for 15 min. Figure 1 shows the atomic arrangements and differential conductance d*I*/d*V* spectra of WS_2_ at 77 K in an ultrahigh vacuum (UHV) environment.

### STS Measurements

STS measurements were carried out using the lock-in technique (bias modulation δV = 5–10 mV, *f* = 700–900 Hz). Using this lock-in technique, standard d*I*/d*V* vs *V* spectra as well as topographic differential conductance d*I*/d*V* maps for a fixed set voltage in constant-current mode were acquired. The latter is used to map the surface LDOS information at the energy corresponding to the set voltage and further obtain the QPI information from each point defect on the WS_2_ surface in Figures 2 and 3.

## Supporting Information Available

Detailed information on the substrate-induced moiré pattern in Supporting Figure S1; quantitative band structure analysis of the energy-dependent QPI wavevector and the large spin-splitting characteristic at the Q valley in ML-WS_2_ in Supporting Figure S2; defect number density offering quantitative information on common defect types in this work, such as O_s_^(top)^, O_s_^(bottom)^, Mo_W_, and C_s_^–^, in Supporting Figure S3; effects of tip potentials with the phase behavior (ϕ_*shift*_) of the QPI standing wave in Supporting Figure S4; and energy-dependent landscape of phase shift variation (Δϕ_*shift*_) for all defect types in this work, which can be constructed and quantified by the same analysis process described in the main content, and the detailed fitting results and data profile of C_s_^–^, O_s_^(top)^, O_s_^(bottom)^, and Mo_w_ in Supporting Figure S5 (PDF)
