# Supplementary material for: Atomically Resolved Defect-Engineering Scattering Potential in 2D Semiconductors
Source: ACS Nano. 2024 Jun 26;18(27):17622–9. doi: 10.1021/acsnano.4c02066 (PMC11238616; doi:10.1021/acsnano.4c02066)
Supplement: Supplementary file 1 — nn4c02066_si_001.pdf [file nn4c02066_si_001.pdf]

# Supporting Information

## Atomically Resolved Defect-Engineering Scattering Potential in 2D Semiconductors

Hao-Yu Chen[1], Hung-Chang Hsu[2], Jhih-Yuan Liang[2], Bo-Hong Wu[2], Yi-Feng Chen[1], Chuan-Chun Huang[2], Ming-Yang Li[3], Iuliana P. Radu[3], Ya-Ping Chiu[*1,2,4,5]

[1] Graduate School of Advanced Technology, National Taiwan University, Taipei 10617, Taiwan

[2] Department of Physics, National Taiwan University, Taipei 10617, Taiwan

[3] Taiwan Semiconductor Manufacturing Company, Hsinchu 30078, Taiwan

[4] Institute of Physics, Academia Sinica, Taipei 115201, Taiwan

[5] Institute of Atomic and Molecular Sciences, Academia Sinica, Taipei 106319, Taiwan

# Supporting Information 1

Moiré pattern at energy level near conduction band minimum of $WS_2$

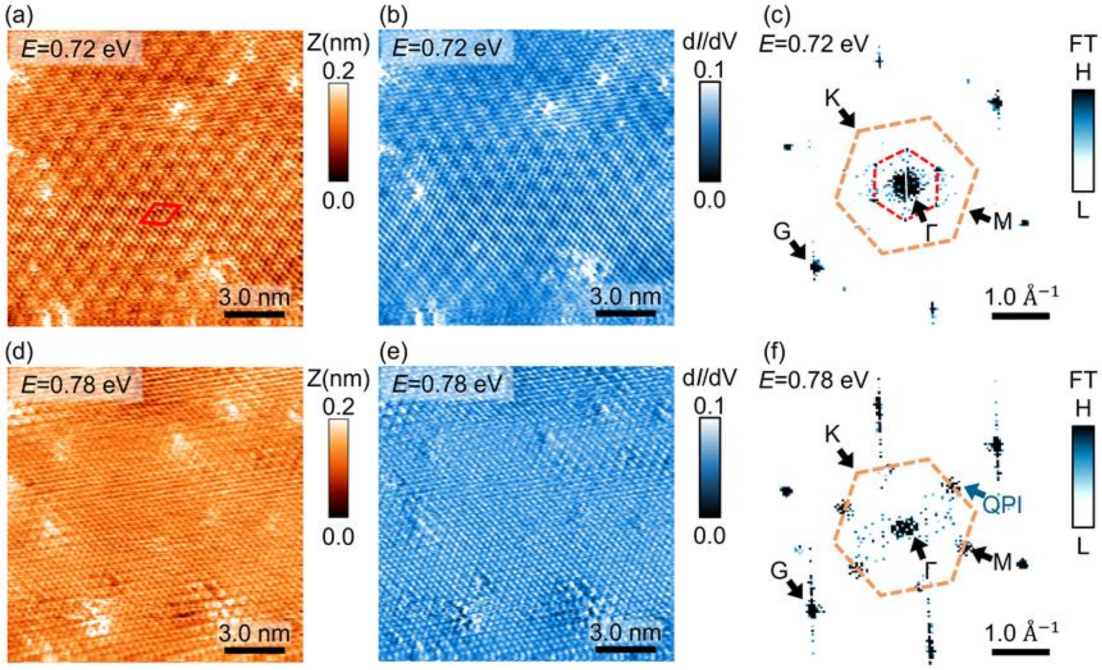


**Figure S1**: **(a, d)** The STM images recorded at energy levels +0.72 eV (below-gap) and +0.78 eV (above-gap) with constant current 100 pA. The moiré unit cell is indicated by the red rhombus. **(b, e)** The d$I$/d$V$ images corresponding to (a) and (d) show the moiré patterns difference between +0.72 eV and +0.78 eV. **(c, f)** The FT-STS maps correspond to (b, e), and the outermost orange dashed hexagon is the 1$^{st}$ Brillouin zone of $WS_2$. The inner red dashed hexagon indicates the moiré pattern due to the lattice mismatch and stacking angle between ML-$WS_2$ and HOPG substrate.

Due to the lattice mismatch and stacking angle between the ML-$WS_2$ and HOPG substrates, the moiré pattern can be observed in the STM and the corresponding d$I$/d$V$ images at the below-gap energy levels of ML-$WS_2$. (See +0.72 eV as an example in **Figure S1 (a)** and **(b)**)

It is noteworthy that the corresponding FT-STS map in **Figure S1 (c)** exhibits a clear moiré period, as marked by the inner red dashed hexagon, but no QPI period is observed at the **M**-point.

However, the moiré pattern disappears when the energy levels lie in the upper gap of ML-$WS_2$, as measured at +0.78 eV in **Figure S1 (d)** and **(e)**. On the contrary, the QPI period can be observed

at the **M**-point in **Figure S1 (f)**.

Since the energy levels of the QPI experimental results in this work lie above the ML-$WS_2$ gap, the moiré patterns do not affect our QPI observations.

# Supporting Information 2

Energy-dependent interference pattern wavevector analysis

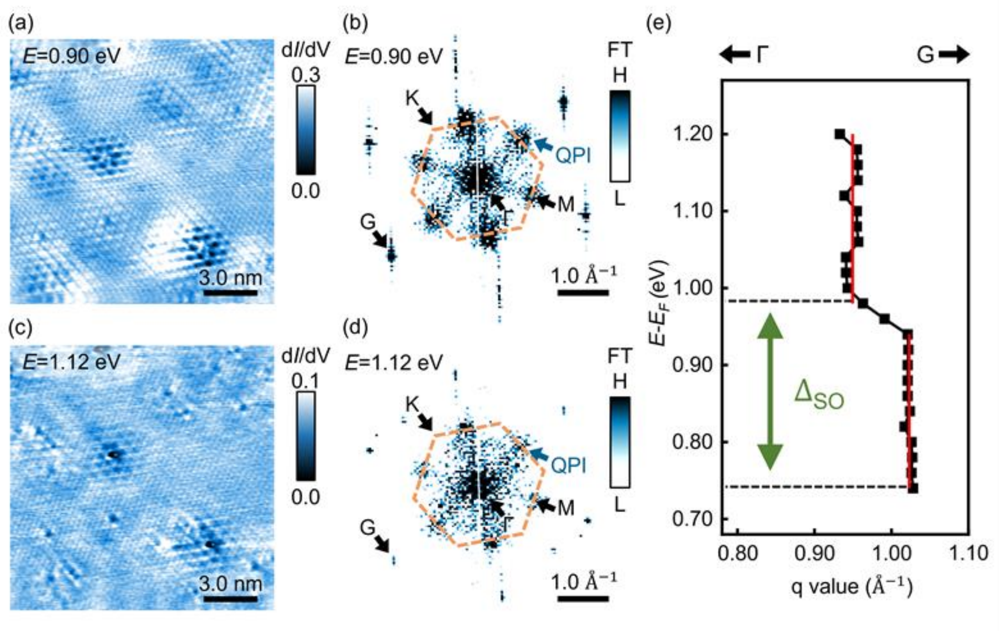


**Figure S2**: **(a, c)** The d$I$/d$V$ images recorded at energy levels +0.90 eV and +1.12 eV at constant current 100 pA. **(b, d)** The +0.90 and +1.12 eV FT-STS maps corresponding to the energy level at the lower and upper spin subband Q valleys, respectively. The orange dashed hexagon is the 1$^{st}$ Brillouin zone of ML-$WS_2$. **(e)** The q-value of each QPI pattern extracted from FT-STS maps with an energy interval between +0.70 and +1.20 eV reveals that the quantity of spin splitting at the Q valley in ML-$WS_2$ is approximately 0.24 eV.

**Figure S2 (a)** and **(c)** are the d$I$/d$V$ images of constant-current measurements recorded at +0.90 eV and +1.12 eV energy levels, and **Figure S2 (b)** and **(d)** are the corresponding 2D Fourier transform results (FT-STS maps). Comparing the FT-STS maps in **Figure S2 (b)** and **(d)**, at energies of +0.90 eV and +1.12 eV, the (2×2)-like signal spot around the **M**-point is preserved at both energy levels, but at +1.12 eV it becomes relatively closer to the center of the **Γ**-point in the FT-STS map. By gradually recording the wave vectors (q-values) of the (2×2)-like period extracted from the FT-STS results in the 0.02 eV energy interval, the energy-dependent evolution of the (2×2)-like period is

shown in **Figure S2 (e)**. Thus, based on the quantitative wavevector analysis at the different energy levels and referred to the previous works,[1,2] the spatially distributed (2×2)-like period near defects is suggested to be the intervalley interference dominated by electron scattering between Q valleys in this work.

Considering that the energetic level of +0.74 eV shows the first presence of the (2×2)-like pattern at the M-point in this work. It could correspond to the energy minimum of the lower spin subband Q valley. In addition, as the energy level continuously increases, the q-value shifts from 1.02 $Å^{-1}$ to 0.95 $Å^{-1}$ at the energy level of +0.98 eV. It could be the energy minimum of the upper spin subband Q valley. Therefore, the spin splitting at the Q valley in ML-$WS_2$ can be estimated to be $\Delta_{SO}$=0.98-0.74 =0.24 eV in this work, confirming the large spin-splitting characteristic at the Q valley in ML-$WS_2$.[1,2]

**Reference:**

[1] Chen, H.-Y., Hsu, H.-C., Huang, C.-C., Li, M.-Y., Li, L.-J. & Chiu, Y.-P. Directly Visualizing Photoinduced Renormalized Momentum-Forbidden Electronic Quantum States in an Atomically Thin Semiconductor. *ACS Nano* **2022**, 16, 9660-9666.

[2] Kormányos, A., Burkard, G., Gmitra, M., Fabian, J., Zólyomi, V., Drummond, N. D. & Fal'ko, V. k· p theory for two-dimensional transition metal dichalcogenide semiconductors. *2D Mater.* **2015**, 2, 022001.

# Supporting Information 3

Statistically calculated density of defects

| Defect density ($cm^{-2}$) | This work | Reference [1-3] |
|---|---|---|
| $O_s^{(top)}$ | $1.35\pm0.55\times10^{13}$ | $10^{12}$~$10^{13}$ |
| $O_s^{(bottom)}$ | $7.60\pm2.76\times10^{12}$ | $10^{12}$~$10^{13}$ |
| $Mo_W$ | $1.30\pm0.73\times10^{12}$ | $10^{11}$~$10^{12}$ |
| $C_s^-$ | $5.10\pm3.28\times10^{11}$ | $10^{9}$~$10^{11}$ * |

* Including carbon-based substitution on sulfur and negatively charge defect

**Figure S3**: The statistically calculated defect number density in this work, including $O_s^{(top)}$, $O_s^{(bottom)}$, $Mo_W$, and $C_S^-$. The defect number density was statistically calculated for ten random areas on the ML-$WS_2$ surface, with a total counting area of about 200 × 200 $nm^2$.

In order to provide further detailed quantitative information about the defect types, in this study, the defect number density was statistically calculated for ten random areas on the ML-$WS_2$ surface, with a total counting area of about 200 × 200 $nm^2$. Quantitatively, we have similar results with previous research work on defect density statistics.[1-3] **Figure S3** lists the data comparing our results with previous work on defect density statistics.

**Reference:**


[1] Wan, Y., Li, E., Yu, Z., Huang, J.-K., Li, M.-Y., Chou, A.-S., Lee, Y.-T., Lee, C.-J., Hsu, H.-C., Zhan, Q., Aljarb, A., Fu, J.-H., Chiu, S.-P., Wang, X., Lin, J.-J., Chiu, Y.-P., Chang, W.-H., Wang, H., Shi, Y., Lin, N., Cheng, Y., Tung, V. & Li, L.-J. Low-defect-density $WS_2$ by hydroxide vapor phase deposition. *Nat. Commun.* **2022**, 13, 4149.

[2] Cochrane, K. A., Zhang, T., Kozhakhmetov, A., Lee, J. H., Zhang, F., Dong, C., Neaton, J. B., Robinson, J. A., Terrones, M., Bargioni, A. W. & Schuler, B. Intentional carbon doping reveals CH as an abundant charged impurity in nominally undoped synthetic $WS_2$ and $WSe_2$. *2D Mater.* **2020**, 7, 031003.

[3] Schuler, B., Lee, J.-H., Kastl, C., Cochrane, K. A., Chen, C. T., Refaely-Abramson, S., Yuan, S., Van Veen, E., Roldán, R., Borys, N. J., Koch, R. J., Aloni, S., Schwartzberg, A. M., Ogletree, D. F., Neaton, J. B. & Weber-Bargioni, A. How Substitutional Point Defects in Two-Dimensional $WS_2$ Induce Charge Localization, Spin–Orbit Splitting, and Strain. *ACS Nano* **2019**, 13, 10520-10534.

# Supporting Information 4

## Discussion of the effects of tip potentials

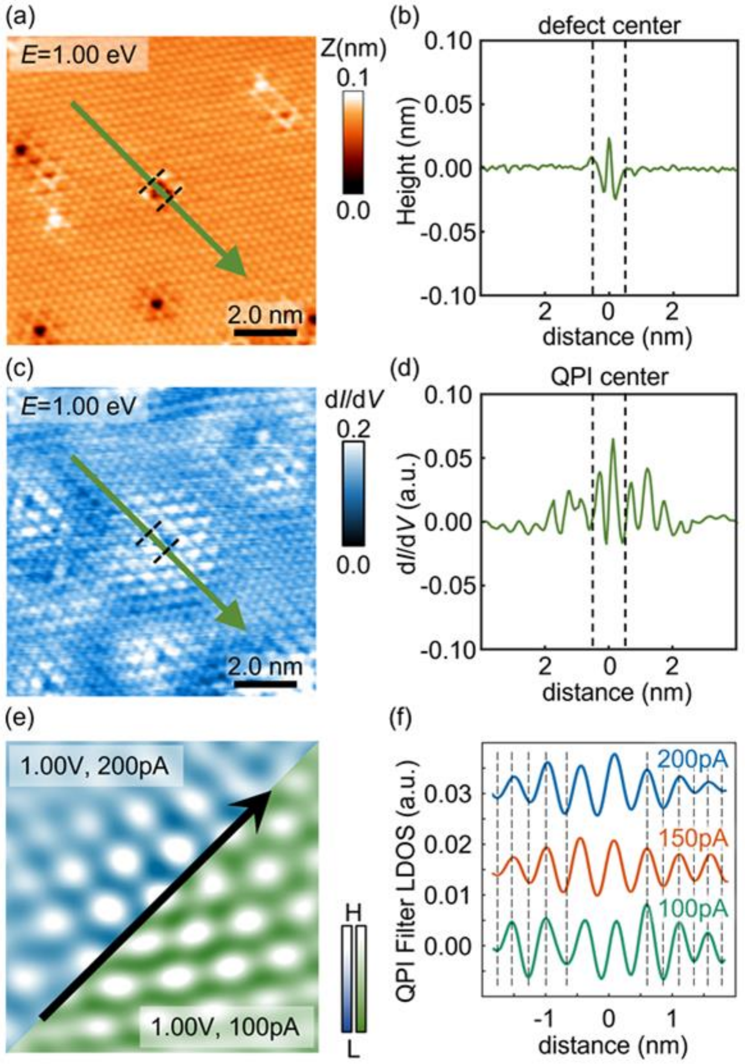


**Figure S4**: **(a)** The 10×10 nm$^2$ STM image recorded at the energy level 1.00 eV. (Set-point: 100 pA) **(b)** The height profiles across the defect, indicating the apparent height variation of the tip-to-sample distance due to the creation of the defect within a distance of < ±0.5 nm centered on the defect. (Highlighted with double black dashed lines) **(c)** The corresponding 10×10 nm$^2$ d$I$/d$V$ image in (a). **(d)** The d$I$/d$V$ data profile in (c) across the defect $C_s^-$ and refers to the green arrow in (c). It shows the spatial distribution of the QPI standing wave is larger than the defect-affected region. **(e)** The QPI patterns enhanced by the 2D-FT filter near defect $C_s^-$ at the same energy levels 1.00 eV with constant current 100 pA and 200 pA. (Image size: 3.5×3.5 nm$^2$) **(f)** The QPI standing wave profile at different constant current set-points, such as 100 pA, 150 pA, and 200 pA. Each profile is extracted along the QPI wavefront propagation direction, as the black arrow indicated in (e). The dashed line indicated the wave peaks and troughs, referenced by the profile at constant current at 100 pA.

(1) First of all, we would like to clarify that the QPI data for this research work has removed the geometric structural height difference due to the presence of defects, as well as the QPI data for the area of this structural height difference. **Figure S4 (a)** and **(c)** are the STM image and the corresponding d*I*/d*V* image recorded at the energy level 1.00 eV, respectively. By taking defect $C_s^-$ as an example, **Figure S4 (b)** presents the height profiles across the defect, indicating the apparent height variation of the tip-to-sample distance due to the creation of the defect within a distance of < ±0.5 nm centered on the defect. (Highlighted with double black dashed lines) Other regions away from the defect (> ±0.5 nm) are relatively flat and stable, and tip height fluctuations are quite small in **Figure S4 (b)**. The phase shift calculation of the QPI standing wave in this work focuses on the data in the tip-height stable region (> ±0.5 nm).

(2) In addition, in order to investigate whether the tip potential affects the phase shift of the QPI standing wave, we varied the distance between the tip and the sample and measured d*I*/d*V*. Technically, varying the distance between the tip and the sample involves modifying the set point of the constant current ($I_{set}$) to vary the distance between the tip and the sample with a fixed sample bias. Experimentally, between 0.85 eV and 0.90 eV, the data show a phase shift of about $2\pi/3$ for the point-defect $C_s^-$ as an example. From 0.85 eV to 0.90 eV (keeping $I_{set}$ = 100 pA), we can use the kappa value (~ 1.05 $Å^{-1}$) to roughly estimate the change in tip-sample distance ΔZ about 0.04 nm.[1] **Figure S4 (e)** shows the comparison of QPI patterns enhanced by 2D-FT filters near the point defect $C_s^-$. The

blue- and green-half figures are obtained at the same energy level of 1.00 eV but with $I_{set}$ = 200 pA and 100 pA, respectively. **Figure S4 (f)** shows the QPI standing wave profiles extracted along the QPI wavefront propagation direction (black arrows in **Figure S4 (e)**) at the same energy level with different $I_{set}$ (from $I_{set}$ = 100 pA to 200 pA) corresponding to different tip-sample distances ($\Delta Z \sim 0.04$ nm). Comparing the experimental data (point defect $C_s^-$ induced phase shift of about $2\pi/3$) with the analytical results in **Figure S4 (f)** due to the tip-sample distance difference, no significant phase behavior difference in **Figure S4 (f)** is observed. Furthermore, the recent theoretical study also has shown that even if the tip-sample distance is changed by 0.2 nm, the phase shift difference is only about $0.1\pi$,[2] which is much smaller than the phase shift variations observed for all types of defects in this study.

Therefore, based on the discussion related to the tip-potential effect, it is suggested that the phase behavior of the QPI standing wave is mainly dominated by defect scattering potential.

**Reference:**

[1] Zhang, C., Chen, Y., Johnson, A., Li, M.-Y., Li, L.-J., Mende, P. C., Feenstra, R. M. & Shih, C.-K. Probing Critical Point Energies of Transition Metal Dichalcogenides: Surprising Indirect Gap of Single Layer $WSe_2$. *Nano Lett.* **2015**, 15, 6494-6500

[2] Kotzott, T., Bouhassoune, M., Prüser, H., Weismann, A., Lounis, S. & Wenderoth, M. Scanning tunneling spectroscopy of subsurface Ag and Ge impurities in copper. *New J. Phys.* **2021**, 23, 113044

# Supporting Information 5

Energy-dependent QPI standing wave phase shift calculations

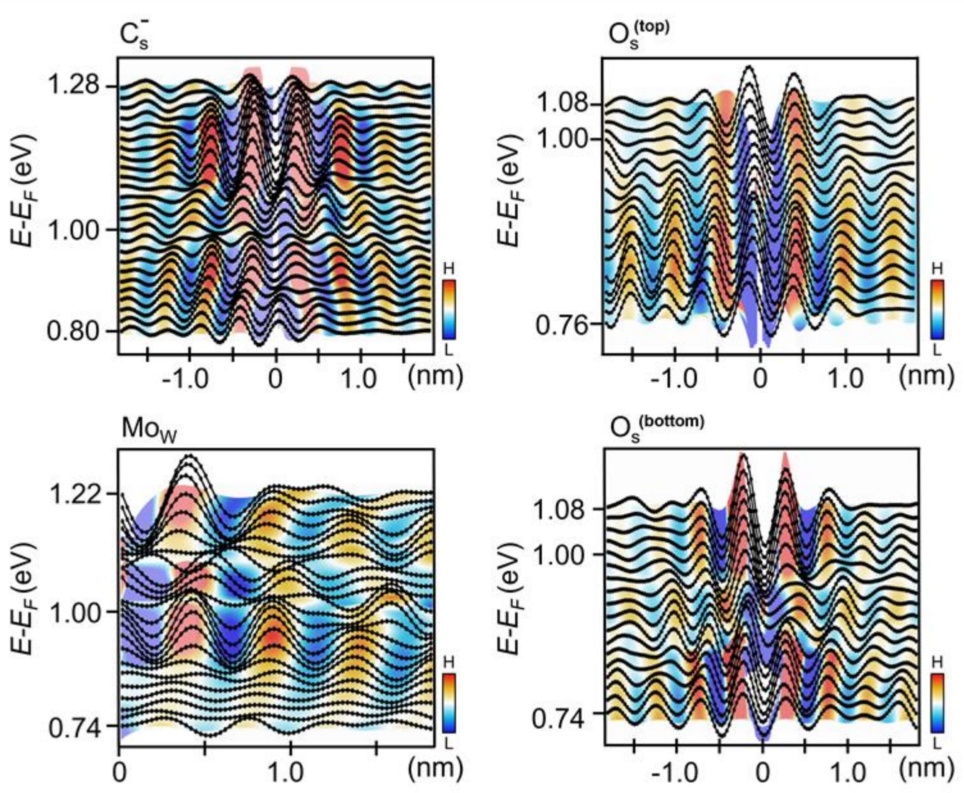


**Figure S5**: The fitting result (red-blue gradient color) and the experimental data (black point profile) of $C_S^-$ , $O_s^{(top)}$, $O_s^{(bottom)}$, and $Mo_W$. The data profiles are extracted from the QPI standing wave enhanced d$I$/d$V$ image along the QPI wavefront propagation. Due to the observed threefold symmetry properties of $Mo_W$, the energy-dependent phase variation analysis is along one single side of the QPI wavefront propagation near $Mo_W$.

The energy-dependent landscape of phase shift variation ($\Delta\phi_{shift}$) for all defect types can be constructed and quantified by the same analysis process described in the main content. The detailed fitting result (red-blue gradient color) and the experimental data of $C_S^-$ , $O_s^{(top)}$, $O_s^{(bottom)}$, and $Mo_W$ are shown in **Figure S5**.
